# Reducing medical student debt: 12 practical tips for innovative service commitment awards

**DOI:** 10.15694/mep.2019.000167.1

**Published:** 2019-09-11

**Authors:** Michelle Schmude, Suzanne McNamara, Roxanne Seymore

**Affiliations:** 1 Geisinger Commonwealth School of Medicine

**Keywords:** medical school debt, service awards, financial aid, logic model, need-based financial aid, merit-based financial aid

## Abstract

This article was migrated. The article was marked as recommended.

At Geisinger Commonwealth School of Medicine (GCSOM), we are developing strategies to reduce the rising debt of our medical students. During a collaborative and iterative process that involved a surprisingly wide group of stakeholders, we developed an innovative service commitment award for medical students: the Abigail Geisinger Scholars Program. Beginning with the spring 2019, GCSOM selected 10 current students for the program, and an additional 10 medical students from the class of 2023 will receive the award beginning in fall 2019. With 20 students enrolled in the Abigail Geisinger Scholars Program, student debt for these students will be decreased by approximately $3.9 million. The potential of this program to significantly reduce medical student debt while repopulating the physician shortage in the primary service areas of Geisinger is tremendous. As the loan debt of medical students continues to rise, medical schools around the country should strategically think about innovative ways to reduce this burden while remaining true to the mission of their institution.

## Introduction

The road to medical school, residency, and practice as a physician is a long and arduous one, but increasing numbers of college graduates are applying to medical school ( Association of American Medical Colleges, 2018). In fact, according to the National Resident Matching Program (NRMP), this year’s residency match was the largest recorded in history with 32,194 first-year (PGY-1) positions ( The National Resident Matching Program, 2019). Although these 32,194 medical school graduates are on their way to becoming practicing physicians, many are faced with an overwhelming amount of medical school debt. For these students, the thought of the repaying graduate school debt (and possibly undergraduate debts as well) is a daunting one. Indeed, students’ debts from medical school continue to receive widespread attention. For example, the Association of American Medical Colleges (AAMC) reported that the median cost of attending medical school and student debt have steadily increased from 2009 to 2016: in 2016 76% of medical school students graduated with educational debts (median amount $190,000) for which monthly repayment were estimated to range from $1500 to $2800 ( Youngclaus, Fresne and Bunton, 2017). Further, the median four-year cost of attendance, which is the total cost of 1 year in medical school, ranged from $213,000 in 2009 to $249,000 in 2016-a 16.9% increase ( Youngclaus, Fresne and Bunton, 2017). Direct costs such as tuition and fees and indirect costs such as living expenses and insurance comprised the greatest portion of these debts. In addition, between 2009 and 2016 the median medical school debt rose from $179,000 to $190,000, a 6.1% increase ( Youngclaus, Fresne and Bunton, 2017). For students who graduated in 2018, the median medical school debt rose to $200,000, an increase of $21,000 from 2009 ( The National Resident Matching Program, 2019). In addition, medical school debt is not distributed uniformly in the United States: According to a 2013 study, among Blacks, Whites, Hispanics, and Asians the percentages of students reporting anticipated debts > $150,000 were 77.3%, 65.1%, 57.2%, and 50.2%, respectively ( Dugger *et al*., 2013).

Realizing that the cost to attend medical school continues to rise and directly affects future physicians’ major life choices (eg, when to start a family, what specialty to choose) ( Rohilfing *et al*., 2014), medical school administrators are thinking strategically about this issue and have begun to implement programs (eg, regarding financial literacy) to educate students about debt. In addition, medical schools are expanding gift aid program (scholarships and awards that do not have to be repaid) and service commitment awards (postgraduate practice in the catchment area of the degree-awarding university in exchange for debt forgiveness) that reduce the costs of education for future physicians. Unfortunately, medical student debt continues to rise, and the paucity of guidances and reports about successful debt-reduction programs challenges everyone involved in medical education. Here we report specific steps that we have taken at our medical school to mitigate student debt and to help meet local health care needs via a novel service commitment award.

Geisinger Commonwealth School of Medicine (GCSOM) is a private allopathic medical school founded in 2009 in Scranton, PA, with a strong commitment to serving local communities and educating physicians to care for these patients. Acknowledging the negative effects of increasing medical student debt, GCSOM has held tuition increases to 2% per year for the past 4 years, but, even so, 82% of our 2018 medical students graduated with a median debt of $256,000 ( Scheinman, 2019). Adding urgency to our attempts to mitigate the adverse effects of medical school debt, we are acutely aware that more than 23% of our medical students are the first in their families to attend university and must borrow to finance their educations ( Scheinman, 2019). To address these needs, we have developed a service commitment award known as the Abigail Geisinger Scholars Program. The goals of the program are 1) to address our medical students’ rising debt and 2) to reduce the physician shortage within the Geisinger service area ( Gil *et al*., 2016). The Abigail Geisinger Scholars Program allows participating students to receive up to 4 years of tuition in the form of a loan that is forgiven as they complete a period of employment as Geisinger physicians equal to the number of years for which they received the award ( Schmude and McNamara, 2019).

Based on our investigations, only limited research and general information are available regarding medical schools that offer self-funded service commitment awards. To share our experiences and learnings, here we present 12 tips to provide guidance to the medical school community regarding novel programs to assist students in reducing their debt upon graduation from medical school. Each tip contains information regarding the lived experiences of those who developed, implemented, and assessed the Abigail Geisinger Scholars Program and the relevant literature that supports the theoretical framework of each tip.

## Tip 1: Establish your team

Members of the GCSOM leadership team realized that the increasing debt of the medical students needed to be addressed but, aside from the normal gift aid approach, really did not know how to begin to tackle such an issue. Therefore, an executive committee met to evaluate the partners needed to begin stakeholder identification and to consider options of debt reduction for our medical students utilizing stakeholder analysis ( Geisinger Commonwealth School of Medicine, 2019; Bryson, 2011).

After an iterative process to determine key constituencies and champions ( Kotter and Rathgeber, 2006), we formed the Abigail Geisinger Scholars Committee with members including the following: President and Dean, Admissions and Financial Aid Dean, Director of Financial Aid, Student Affairs Dean, Chief Financial Officer, Chief Diversity Officer, a GCSOM student, Physician Recruitment/Human Resources, Legal Services, Tax and Accounting, and Physicians. The 12 members of the Abigail Geisinger Scholars Committee met over the course of 12 months to develop and propose the service commitment award known as the Abigail Geisinger Scholars Program to the Geisinger Executive Leadership team and the Geisinger Board of Directors ( Kotter, 2012).

## Tip 2: Know your project: gather all your facts

At the outset, the Abigail Geisinger Scholars Committee thoroughly assessed the issues and conducted research to collect the necessary information. Because reducing medical student debt is a strategic initiative at GCSOM, we felt it was imperative to invest the time to understand the problem and come to consensus about how we would address it. Specifically, we first agreed that a service commitment award would address the problem of student debt while allowing GCSOM to create a physician pipeline into the health system. During our research we identified the US military-based Health Professional Scholarship Programs (HPSP) and the National Health Service Corps Service commitment programs. We discovered that each program offered both scholarship and loan repayment forgiveness programs for students who meet stated criteria in exchange for a service commitment ( US Department of Health and Human Services, 2019).

In addition, the committee agreed to contact the AAMC to determine if other medical schools offered any type of loan forgiveness or service commitment programs. At that time, the AAMC did not have any information regarding medical schools and service commitment programs (personal communication, February, 2018). During our research, we identified the First 5 Riverside Medical Student Service Award Scholarship Program at the University of California Riverside ( Epstein, 2018). This program is designed for students who have completed 5 years of pediatric practice in Riverside county, California ( University of California Riverside, 2019). The committee agreed that a service commitment award offered by a medical school was an innovative strategy to reduce medical student debt and bring future physicians into the Geisinger system.

## Tip 3: Develop relationships within your tribe

Because our School of Medicine integrated into Geisinger in January 2017, leaders realized that we had to build relationships across a diverse health care system in which many of the key stakeholders had never worker together prior to the committee assignment. This step is critical in the process because relationships, in addition to excellent leadership, build the foundation for effective and efficient group work ( Useem, 2011). By aligning our resources, establishing goals, and building or extending relationships, we leveraged our human resources to advance the strategic initiative of developing a model to reduce medical student debt ( Bolman and Deal, 2013).

## Tip 4: What information do we need from our applicants? Be creative with your documents

In the United States, the federal government regulates student loan borrowing. The Truth in Lending Act (TILA) of 1968 was designed to standardize the required disclosures, terms, and costs associated with borrowing. We learned about the federal guidelines one must follow for program compliance ( U.S. Department of Treasury, 2019). In this context we note that consulting with external and internal legal counsel will ensure both compliance with the TILA regulations and that the committee is following institutional policies and procedures.

After the scholarship committee identified the forms and disclosures that would be required, it created documents that aligned with the program goals. After an exhaustive analysis of required documents, the committee agreed upon the following items: 1) a GCSOM loan agreement, 2) three disclosure forms, and 3) an amortization schedule. Customized loan packets that included these items were created for each student who received the Abigail Geisinger Scholars Award. These documents were vetted through the finance department within our health care system and were reviewed by legal counsel for quality assurance. This step is required to ensure compliance with all federal and internal requirements.

## Tip 5: Solicit feedback

Realizing this was a new and innovative program for GCSOM and in the larger community of medical schools, we felt it was vital to understand any questions or concerns of potential borrowers before we released it to the marketplace. To elicit this feedback, we hosted focus groups with students and presented to staff and faculty to solicit feedback about the strengths and areas of improvement for the program. We emphasize this stage because we believe that education and related services must be developed for and with the consumer. After we gathered and evaluated the information received from our students and the college community, the Abigail Geisinger Scholars Committee made final recommendations to the Geisinger Executive Leadership Team and the Geisinger Board of Directors, and the Board approved implementation beginning in the spring 2019 semester.

## Tip 6: Dress rehearsal: check and double-check

At this stage, the committee developed a detailed time line with established deliverables such a presentation regarding the program and the loan agreement. Buying into shared responsibilities listed in the time line further advances the team concept and holds all accountable for successful implementation. Here are typical questions to ask your team to ensure the project is ready:

• When do specific components need to be ready (ie, what are the deliverables), and what groups must interact and cooperate to make the project successful? Setting reasonable deadlines prioritizes group work that must be accomplished and helps everyone understand the process.
• Should we pilot the program? We knew we wanted to roll out the Abigail Geisinger Scholars Program for the new academic year, but the time line made it clear that we did not have sufficient time to do it successfully. We also knew we did not want to wait an entire year before taking steps, so we decided to pilot the program and release it during the spring 2019 semester. Information and feedback received from this group of students allowed us to make the necessary in-process adjustments for the dissemination of information to the Doctor of Medicine class of 2023.

## Tip 7: Create a process to select the best candidates

In order to select the best candidates for admission into the Abigail Geisinger Scholars Program, we established a smaller team with members who had the appropriate skill sets for this stage; we sought perspectives from the School of Medicine and the Geisinger system, including several physician leaders ( Useem, 2011). The selection committee developed the application requirements, including the following:

• Abigail Geisinger Scholars application
• Applicant’s curriculum vitae
• Applicant’s authorization to review his or her *American Medical College Application Service* application
• Applicant’s letter(s) of recommendation
• Applicant’s essay discussing what the scholarship would mean to them regarding the advancement of their professional goals
• A private education loan applicant self-certification form ( Schmude and McNamara, 2019).

In addition, the team constructed an evaluation rubric so that students who applied for the Abigail Geisinger Scholars Program would be evaluated fairly and consistently ( McConnell, Horan, Zimmerman, and Rhodes, 2019). Criteria contained in the rubric included merit, financial need, and the likelihood of practicing in Geisinger’s regionally preferred areas beyond the service obligation. After an iterative process of seeking feedback and revising accordingly, the Abigail Geisinger Scholars rubric was approved by all constituencies and then was used in the review of candidates who applied to the program. Ultimately, this rubric assisted the selection committee in the alignment of evaluation criteria to the goals of the program, thereby making the review process effective and efficient.

## Tip 8: Creative ideation: building an integrated marketing communications plan

You can have a great product but if no one knows about it or if they are not your target audience, you may never realize your goals ( Rodgers and Thorson, 2013). Therefore, we established an integrated marketing communications campaign to let our audience (suitable medical school students) know about our product (the Abigail Geisinger Scholars Program) by creating attention, interest, desire, and action ( Altstiel and Grow, 2013). First, we developed a time line for the launch of the program. We developed a press release that was sent to all local media outlets, and we received an overwhelming response of free publicity. Next, we collaborated with our internal integrated marketing communications division to develop a logo, brochure, and website for the program. After the selection committee chose the candidates, a photo shoot was scheduled and a press release was created and sent to all identified media outlets. This then resulted in various articles in newspapers and interviews on network television for those selected into the program. Finally, we have reported or will report various aspects of the program at local and national conferences, with positive feedback ( Schmude, 2018).

## Tip 9: Ready, set, go live! Roll out to students

Because our program, in the beginning stages, was offered to both new and current students, we needed to present the information and content in different ways. We began by holding informational sessions for our current students. These comprised several speakers, which allowed students the opportunity to understand the program from several different aspects. Financial Aid kicked off the session with a program overview, time line of events, and deadlines for applicants. Experts in the areas of Human Resources and Finance discussed physician placement within the Geisinger System, loan repayment, loan forgiveness, tax implications, and amortization schedules. Attendance was mandatory for those applying, which gave us the opportunity to use the session in a manner similar to that of Entrance Counseling for federal loan borrowers (see https://studentloans.gov).

For new students, we offered the sessions via Zoom, a web-based conferencing and messaging application that was available at different times and allowed maximum attendance for students interested in the program for the 2019-2020 academic year. Zoom was used so that students could attend the sessions virtually and not have to incur additional expenses to travel to GCSOM for in-person presentations. Potential applicants were able to ask questions in real time, and the sessions were recorded for future student events. During the question and answer session, our team took notes of trends so that we could best address questions and concerns for future presentations. Based on information gleaned from these sessions, we have added a Frequently Asked Questions section to our website for future applicants to view.

After reviewing feedback from these sessions, we made a few revisions to our time lines and deadlines for next academic year to best serve our students. During the sessions, we received a few student questions that we did not anticipate, so we learned to ensure that we had access to the correct people in our organization for timely responses to a wide range of questions. For these reasons, we needed to put into place a process for handling questions that came into the Financial Aid office but required the attention of someone in the health care system. We decided it was best to create a core team from the Geisinger System, including Financial Aid, Human Resources, Legal, and Finance. This core team’s ongoing activities including fielding questions and retrieving answers in a timely manner. This core group meets monthly to discuss issues ranging from form changes to process improvements.

## Tip 10: Develop an effective and efficient applications review process

After a student submits an application packet, the review process begins. Each packet is manually reviewed to ensure all documents are received, signed, and complete. Once we have a completed packet, the Financial Aid office uses the rubric to score each applicant. The packet is then redacted to remove personally identifiable information, including name, address, phone number, college attended, and sex, before forwarding to our selection committee for review and placement scoring. To eliminate bias, the committee reviews the applications blindly and awards 10 Abigail Geisinger Scholars and 5 alternatives. Alternates are selected to replace students who decide to forego the program. For the 20 awards offered thus far, we have not needed to offer the award to the alternates. Based on the 100% acceptance rate, students who apply and are offered the award, accept it.

The Financial Aid office creates customized notification letters, disclosures, and contracts for each student, signed by our Associate Dean of Admissions, Enrollment, and Financial Aid. The financial aid office revises each candidate’s financial aid package to reflect the award and loan amounts accordingly. Typically, most student are eligible only for the Federal Direct Unsubsidized loan, and unless the student states otherwise, they can seek alternative resources (ie, private loan).

The financial aid office personally meets with each selected candidate to discuss how the program is awarded and how much the student is borrowing, and candidates are given a deadline by which to submit their signed loan agreements and disclosures. The loan agreement is then signed by the director of Financial Aid and the associate vice president of Human Resources; the original document is held in the office of Human Resources. Financial Aid receives a copy of the finalized document for the student’s financial aid folder.

## Tip 11: Look back—what went right and what needs improvement

All successful programs should be evaluated and assessed for effectiveness, and the Abigail Geisinger Scholars Program is no different. We created a brief student survey (a few easy-to-answer questions) asking students for feedback and critiques. The results of the survey were shared in an executive summary with our leadership for their thoughts, follow-up, and action. Because this program is in its infancy, we are continuing our monthly team meetings to assess feedback and suggestions, allowing the program to grow.

## Tip 12: Plan for the future

Plans are already under way for the MD class of 2024. We have instituted monthly meetings so all constituencies know the progress of the students who are Abigail Geisinger Scholars and can be prepared for the class of 2024. In addition, we hope to expand the number of scholars in the future. Finally, we will review potential areas for further research and advance our continuous quality improvement.

## Conclusion

How much can we expect medical students to borrow before the cost of medical school deters qualified candidates from taking care of the aging population of the United States? This question must be addressed as we face a looming physician shortage and rising debt of medical school graduates. By engaging in a collaborate process to identify strategic ways to reduce medical student debt, we can address this growing problem. At GCSOM, we developed the Abigail Geisinger Scholars Program to significantly reduce the debt of our medical students by approximately $2.26 million a year or more than $9 million in total once we have 10 students per class in the program. If it were to expand, we could see an exponential decrease in our students’ debt.

We stress that open communication with partners paired with an evaluation process that implements change in real time are the keys to success with a complex initiative such as the Abigail Geisinger Scholars Program. In the future, we hope to offer more awards to deserving students who will care for people who live within the Geisinger clinical delivery area-the reason for the founding of Geisinger Commonwealth School of Medicine.

## Take Home Messages

• For 2018 graduates, median US medical school debt rose to $200,000, an increase of $21,000 from 2009, and medical school debt is not distributed uniformly (eg, by racial or economic background).
• We report 12 specific steps we are taking to mitigate student debt and help meet local health care needs via a novel service commitment award.
• We offer specifics about identifying and involving important stakeholders across undergraduate medical education and a clinical delivery system.
• Twenty medical students have received Abigail Geisinger Scholars awards that are reducing our medical students’ debt by approximately $2.26 million per year.

## Notes On Contributors

**Michelle Schmude,** EdD, MBA, Associate Dean for Admissions, Enrollment Management and Financial Aid, and Associate Professor, Department of Medical Education, Geisinger Commonwealth School of Medicine. ORCID iD: https://orcid.org/0000-0002-0837-5355

**Suzanne McNamara,** BS, Director of Financial Aid **,** Geisinger Commonwealth School of Medicine, Geisinger Commonwealth School of Medicine.

**Roxanne Seymour,** MBA,Associate Director of Financial Aid **,** Geisinger Commonwealth School of Medicine, Geisinger Commonwealth School of Medicine.

## References

[ref1] Altstiel T and Grow J. (2013) Advertising Creative Strategy, Copy, Design. Thousand Oaks, CA: Sage Publishing

[ref2] Association of American Medical Colleges.(2018) Medical student education: debt, costs, and loan repayment fact card. Available at: https://store.aamc.org/downloadable/download/sample/sample_id/240/( Accessed: 02 July 2019)

[ref3] Bolman L. and Deal T. (2013) Reframing Organizations. San Francisco, CA: Jossey-Bass

[ref4] Bryson J. (2011) Strategic Planning for Public and Nonprofit Organizations. San Francisco, CA: Jossey-Bass

[ref5] Dugger R. A. El-Sayed A. M. Dogra A. Messina C. (2013) The color of debt: racial disparities in anticipated medical student debt in the United States. PLoS One. 8(9),e74693. 10.1371/journal.pone.0074693 24019975 PMC3760863

[ref6] Epstein E. (2018) A California city’s plan to turn indebted millennials into local doctors, Politico. Available at: https://www.politico.com/magazine/story/2018/01/18/riverside-california-what-works-millennial-doctors-216475( Accessed: 02 July 2019)

[ref7] Geisinger Commonwealth School of Medicine.(2019) Abigail Geisinger Scholars Program. Available at: https://www.geisinger.edu/education/admissions/financial-aid/scholarships-grants/abigail-geisinger-scholars-program( Accessed: 02 July 2019)

[ref8] Gil J. A. Waryasz G. R. Liu D. and Daniels A. H. (2016) Influence of medical student debt on the decision to pursue careers in primary care. Rhode Island Medical Journal. 99(7), pp19–21 27379353

[ref9] Kotter J and Rathgeber H. (2006) Our Iceberg Is Melting. London: Pan Macmillan

[ref10] Kotter J. (2012) Leading Change. Brighton, MA: Harvard Business Publishing 10.15358/9783800646159

[ref11] McConnell K. K. Horan E. M. Zimmerman B. and Rhodes T. (2019) We Have a Rubric for That: The VALUE Approach to Assessment. Washington, DC: Association of American Colleges and Universities

[ref12] National Resident Matching Program.(2019) Thousands of residents celebrate NRMP match results. Available at: http://www.nrmp.org/one-nine-press-release-thousands-resident-physician-applicants-celebrate-nrmp-match-results/( Accessed: 02 July 2019)

[ref13] Rodgers S. and Thorson E. (2013) Advertising Theory. New York, NY: Routledge

[ref14] Rohilfing J. Navarro R. Maniya O. Z. Hughes B. D. (2014) Medical student debt and major life choices other than specialty. Medical Education Online. 19, p.24603. 10.3402/meo.v19.25603 PMC4229497 25391976

[ref15] Scheinman S. J. and Ryu J. (2019) Why a teaching hospital offers an employment-based tuition waver program, NEJM Catalyst. Available at: https://catalyst.nejm.org/teaching-medical-students-tuition-waiver/( Accessed: 02 July 2019)

[ref16] Schmude M. (2018) Reducing student debt through the creation of a service commitment/loan forgiveness program. Tabletop session: AAMC National General Meeting.(03 November), Austin, TX. https://custom.cvent.com/127DD37CC17748578EE776BCD2E2C949/files/63d9129b622a44118c464b1257b6915a.pdf

[ref17] Schmude M. and McNamara S. (2019) Reducing student debt through the creation of a service commitment/loan forgiveness program. Presented at: AAMC Health Professions Financial Aid Administrators Conference.(06 February), Savannah, GA. http://www.cvent.com/events/2019-health-professions-financial-aid-administrators-conference/agenda-e8c80431a3224f1e9ba3b5eb89bbaad6.aspx

[ref18] University of California Riverside.(2019) Scholarship opportunities. Available at: https://somsa.ucr.edu/scholarship-opportunities( Accessed: 02 July 2019)

[ref19] US Department of Health and Human Services.(2019) National Health Service Corps (NHSC). Available at: https://www.usphs.gov/student/nhsc.aspx( Accessed: 02 July 2019)

[ref20] US Department of Treasury.(2019) Truth in lending. Available at: https://www.occ.gov/topics/consumer-protection/truth-in-lending/index-truth-in-lending.html( Accessed: 02 July 2019)

[ref21] Useem M. (2011) The Leader’s Checklist. Philadelphia, PA: Wharton Digital Press

[ref22] Youngclaus J. Fresne J. and Bunton S. A. (2017) An updated look at attendance cost and medical student debt at U.S. medical schools. AAMC Analysis in Brief. 17(1), pp.1–3

