# Supplementary material for: Reducing medical student debt: 12 practical tips for innovative service commitment awards
Source: MedEdPublish (2016). 2019 Dec 13;8:167. Originally published 2019 Sep 11. [Version 2] doi: 10.15694/mep.2019.000167.2 (PMC10712507; doi:10.15694/mep.2019.000167.2)
Supplement: Peer review history

# Reviewer report: Reviewer response for version 2

**DOI:** 10.21956/mep.20252.r31421

**Published:** 2019-12-30

**Authors:** P Ravi Shankar

**Affiliations:** 1 American International Medical University

This review has been migrated. The reviewer awarded 4 stars out of 5 The revised version of the manuscript examines the debt-forgiveness program at a medical school in the United States and the authors offers twelve tips for educational leaders interested in starting similar programs at their home institution. Some of the tips may be more relevant to the United States while others are applicable to a wider context. Details of the chosen scholars and how they compare to the general student population will be of interest. A description of the specific criteria followed in selecting the scholars will also be of interest. This is an important article for medical educators especially considering the rising cost of medical education in most locations.

Reviewer Expertise:

NA

No decision status is available

# Reviewer report: Reviewer response for version 2

**DOI:** 10.21956/mep.20252.r31420

**Published:** 2019-12-13

**Authors:** Trevor Gibbs

**Affiliations:** 1 AMEE

This review has been migrated. The reviewer awarded 3 stars out of 5 Thank you for re-submitting this well written and well structured paper. I am still of the opinion that it covers a very important subject, that of student debt.I feel that the 12 tips described are very informative, probably from a more organisational management approach than an educational one. On reading this second version however, I still feel that I am missing several important points:How transferable is this model to a less well resourced country who are just as desperate for the development of more doctors, but who have greater problems with student debt?- The number of students is a small proportion of the total number I imagine with such debt, which makes selection of the students very important. Although the paper describes the how process it does not describe the descriptive characteristics of the chosen few.- What were the socio-demographic profiles of the chosen students- did they match the statement made earlier n the paper that talks about race and economic factors?- How does the Social Accountability of the school match the chose students ?- What are the opinions of those students who are not chose and how are the scholars seen by their peers who are in great debt ?A useful paper that raises important questions

Reviewer Expertise:

NA

No decision status is available

# Reviewer report: Reviewer response for version 1

**DOI:** 10.21956/mep.19127.r27615

**Published:** 2019-10-17

**Authors:** Trevor Gibbs

**Affiliations:** 1 AMEE

This review has been migrated. The reviewer awarded 3 stars out of 5 Before reading this paper I wasn't that sure what service commitment awards were, and after reaing the paper, that is still not clear. However I did enjoy reading the paper and although it was very US centric I am sure that many other curriculum leaders could learn from reading the paper. I thought that the various tips were well described and informative. However I was still left with an uncertainty of what type of students were taken into this programme, what was their diversity picture and how are they getting on. Presumably these scholarships are for those students who would not normally apply to medical school and logically would end up with greater debt. Also the word commitment does infer that there is something tagged onto these awards that ensure some degree of service post graduation?

Reviewer Expertise:

NA

No decision status is available

# Reviewer report: Reviewer response for version 1

**DOI:** 10.21956/mep.19127.r27616

**Published:** 2019-09-19

**Authors:** Michelle McLean

**Affiliations:** 1 Bond University

This review has been migrated. The reviewer awarded 4 stars out of 5 Thanks for this well-written article. I am sure that this initiative has and will provide relief to some of the medical students. I am not 100% sure what the criteria are to be successful to be awarded a scholarship as the tips says 'good' students which is not defined. My assumption is that this relates to academic achievement, which is usually associated with 'scholarship'. The words 'scholarship' and 'bursary' are often used interchangeably but there is a difference. Scholarships, as the name suggests, reward good scholars so are usually on the basis of academic merit and suitability for the course or research post. Bursaries on the other hand are based primarily on need and so candidates are required to provide financial disclosure so that this need can be assessed. Candidates do need to have a good (not necessarily excellent) academic record as historical social and economic disadvantage may have been a factor in them not reaching their true potential. My experience with regard to bursaries is that there is generally a requirement to work off the debt. What is described as a scholarship in this submission appears to be a mix between a scholarship (academic) and a bursary (work to repay). With the School having a ethos of social responsibility, there is no mention of a commitment to community as a criterion for the 'scholarship'. Is this a consideration in applying for the award?

Reviewer Expertise:

NA

No decision status is available

# Reviewer report: Reviewer response for version 1

**DOI:** 10.21956/mep.19127.r27617

**Published:** 2019-09-12

**Authors:** Linda Nield

**Affiliations:** 1 West Virginia University School of Medicine

This review has been migrated. The reviewer awarded 4 stars out of 5 Dear Authors, Thanks for providing a solution to a huge problem that discourages so many students. I have two questions:1. I'm not sure what this sentence in the introduction means: "Further, the median four-year cost of attendance, which is the total cost of 1 year in medical school, ranged from $213,000 in 2009 to $249,000 in 2016—a 16.9% increase (Youngclaus, Fresne and Bunton, 2017)." Please re-write. 2. Can you explain how loan forgiveness happens exactly? Where does the money come from?I appreciate the information.

Reviewer Expertise:

NA

No decision status is available
